# A Summary of Lightpipe Radiation Thermometry Research at NIST

**DOI:** 10.6028/jres.111.002

**Published:** 2006-02-01

**Authors:** Benjamin K. Tsai

**Affiliations:** National Institute of Standards and Technology, Gaithersburg MD 20899-8441

**Keywords:** blackbody, effective emissivity models, heat-pipe blackbody, lightpipe radiation thermometer, radiation thermometer, temperature, thin-film thermocouple, traceability, uncertainty

## Abstract

During the last 10 years, research in light-pipe radiation thermometry has significantly reduced the uncertainties for temperature measurements in semiconductor processing. The National Institute of Standards and Technology (NIST) has improved the calibration of lightpipe radiation thermometers (LPRTs), the characterization procedures for LPRTs, the *in situ* calibration of LPRTs using thin-film thermocouple (TFTC) test wafers, and the application of *model-based* corrections to improve LPRT spectral radiance temperatures. Collaboration with industry on implementing techniques and ideas established at NIST has led to improvements in temperature measurements in semiconductor processing. LPRTs have been successfully calibrated at NIST for rapid thermal processing (RTP) applications using a sodium heat-pipe blackbody between 700 °C and 900 °C with an uncertainty of about 0.3 °C (*k* = 1) traceable to the International Temperature Scale of 1990. Employing appropriate effective emissivity models, LPRTs have been used to determine the wafer temperature in the NIST RTP Test Bed with an uncertainty of 3.5 °C. Using a TFTC wafer for calibration, the LPRT can measure the wafer temperature in the NIST RTP Test Bed with an uncertainty of 2.3 °C. Collaborations with industry in characterizing and calibrating LPRTs will be summarized, and future directions for LPRT research will be discussed.

## 1. Background

Recently, many advances have been made in semiconductor metrology, as evidenced by the goal in the International Technology Roadmap for Semiconductors (ITRS) to achieve 45 nm dynamic random access memory (DRAM) ½ pitch by 2010 [1]. An important growing sector of this industry has been the rapid thermal processing (RTP) for chemical vapor deposition, physical vapor deposition, oxidation, annealing, silicidation, and oxide-etch processes. Compared to the traditional batch processing of silicon wafers, single-wafer RTP offers advantages of higher ramp rates, shorter processing times, tighter ambient control, and shorter cycle times.

Accurate temperature measurement and control during RTP by using noncontact techniques such as light-pipe radiation thermometers (LPRTs) is crucial for achieving high throughput and maintaining quality. However, achieving accurate traceable temperature measurements by using noncontact LPRTs has been challenging. First, stray light from the source bouncing off reflective surfaces can provide extraneous unwanted signal into the radiometer. Second, temperature variations with time and with wafer location can complicate the measurement process and can increase the uncertainty in the measurements. Third, LPRT temperature measurements can be affected by changes in the wafer’s optical properties, which can vary with temperature, wavelength, wafer location, surface topography, and chemical composition. Fourth, the effective emissivity of the wafer and surroundings, which accounts for interreflections with other surfaces in the radiation chamber, is highly dependent on the geometry and radiative properties of these surfaces. Finally, establishing traceability for LPRT measurements is a nontrivial and significant investment in time and effort.

Implicit in the mission of the National Institute of Standards and Technology (NIST) [2] and of our RTP temperature project is the task of developing high-quality measurement standards and establishing a calibration system whereby users can derive their temperature traceability. To this end, we are committed to establishing a national protocol for calibration of LPRTs using stable blackbodies in the temperature range of 700 °C to 1000 °C traceable to the International Temperature Scale of 1990 (ITS-90) [3].

In the early 1980s at the National Bureau of Standards (now NIST), a fiber-optic lightpipe (LP) coupled to a radiation detector was first used as a thermometer to measure the temperature of gases. The fiber tip exposed to the hot gases was coated with opaque, black films of platinum or rhodium [4–6] to form a cavity, which emitted near-blackbody radiation that was transferred to the radiation detector. In the early 1990s, the ripple technique [7] for RTP applications used rod-type LPs as transfer optics to collect and transfer spectral radiance from wafer targets onto the radiation detector. Since that time, the LPRT has been used to monitor wafer temperature in high-temperature semiconductor processing, because of its minimal thermal disturbance to the heated wafer and the radiation field, and because of the convenience of its noncontact measurement capability [8]. LPRTs are presently used for temperature measurement in rapid thermal annealing, rapid thermal oxidation [9], and rapid thermal chemical vapor deposition [10], as well as for emissivity determinations [11].

During this past decade, NIST has led the effort in assisting industry to characterize the performance of industrial LPRTs, gain an increased understanding of the importance of traceability to a national standard, and develop a greater appreciation for the need for accuracy. Accordingly, the NIST RTP Temperature Project set a goal of achieving 2 °C measurement uncertainty at 1000 °C in temperature accuracy, as outlined in the Semiconductor Industry Association roadmap [1]. This low uncertainty has been accomplished through the four-pronged approach of the NIST RTP temperature project: (1) develop procedures to fabricate and calibrate thin-film thermocouple (TFTC) wafers for *in situ* calibration of the LPRT against a thin-film thermocouple test wafer [12–31]; (2) characterize LPRTs [32–37,79]; (3) develop analytical models to predict the corrections to spectral radiance temperatures using an LPRT calibrated against a blackbody [38–52]; and (4) collaborate with equipment, device, and instrument manufacturers in implementing new methods for reliable and traceable temperature measurements [53–57]. As a result of this effort, in the last ten years at NIST, we have achieved several significant milestones in our noncontact temperature research with LPRTs: (1) calibration of LPRTs using the sodium heat-pipe blackbody (Na-HPBB) with an uncertainty of 0.2 °C (*k* = 1) [29]; (2) temperature measurement using LPRTs and effective emissivity models, resulting in an uncertainty of 3.5 °C [29]; (3) *in situ* calibration of a LPRT using a thin-film thermocouple wafer with an uncertainty of 2.1 °C [29]; (4) qualitative and quantitative optical visualization techniques for evaluating and inspecting sapphire lightpipes [35]; and (5) recommendations for making more accurate temperature measurements using LPRTs [34]. While the work has specifically addressed a semiconductor application, the approaches have general applicability for achieving reliable, traceable temperature measurements using LPRTs in other material processing and manufacturing environments, such as those used in the production of steel, aluminum, and glass.

The purposes of this paper are to document in one place all LPRT work done at NIST in the past decade, reference all NIST papers published about LPRT research, and report on the state of the art in LPRT research. This paper will summarize the types of LPRTs, their calibration and characterization techniques, in situ calibration of LPRTs using TFTCs, and model-based corrections of LPRT measurements. Finally, potential future work in LPRT research is discussed.

## 2. Radiation Thermometers and LPRTs

Traditionally, lightpipe (LP) sensors are attractive in temperature monitoring applications for at least five reasons. First, the noncontact and nondestructive nature of LPs does not alter or destroy the original surface. Second, LPs provide immunity from shock, vibration, and other adverse environments, such as chemical, thermal, and electromagnetic interference. Third, LPs are very convenient especially in confined areas and can be placed very close to a target if desired. Fourth, LPs are safe even in high voltage areas and in ionizing plasma fields. Fifth, high numerical apertures in LPs can reduce significantly the effects of the variability in optical properties [55].

**Fig. 1:**
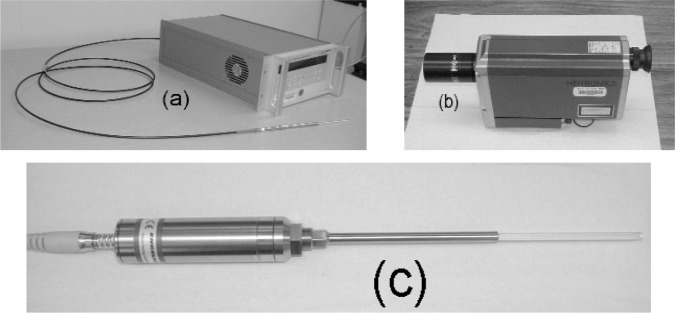
(a) lightpipe radiation thermometer, (b) spot-type radiation thermometer, and (c) cableless lightpipe radiation thermometer.

LPRTs, such as the one in Fig. 1a, typically consists of a high-quality sapphire crystal rod (LP) enclosed in a concentric sapphire sheath and linked by flexible quartz fibers to a silicon detector with a near-infrared filter. Besides the detector, the control box contains the front panel display and the optics and electronics necessary to digitize the measured signal and to convert it into the appropriate radiance temperature. The sapphire rod is enclosed in a concentric sapphire sheath for protection from shock and vibration. The LPs used in our studies are of varying lengths, but they are all approximately 2 mm in diameter. The sapphire sheath that surrounds the LP has a typical outer diameter of approximately 4 mm. In normal operation for measuring the radiance temperature, the LPs are connected to a 1 mm diameter quartz fiber-optic cable. From now on, LPs will refer only to the crystal rod and sheath, while LPRTs will refer to the complete system including the LP rod and sheath, fiber-optic cable, optics, electronics, and all other necessary accessories (excluding the computer and data acquisition system) for measurement of the radiance temperature.

Spot-type radiation thermometers (STRTs) are also becoming common as temperature sensors in RTP applications, especially in areas where it is not possible or feasible to place the LP close to the wafer but there is adequate optical access to the wafer spot. To view the wafer spot, the STRT must have a sufficiently small field-of-view and spot size. A STRT, like the one in Fig. 1b, usually consists of the lens, optics, electronics, eyepiece, and the front panel display. STRTs have been calibrated and researched at NIST. American Society for Testing and Materials (ASTM) standards have been developed for STRTs [56-57], and numerous studies of STRTs have been published [4–8,12,55,58–66].

Cableless lightpipe radiation thermometers (CLRTs) are a new generation of LPRTs, which have all of the attractive features of LPRTs. In addition, the elimination of the flexible cable reduces the measurement uncertainty by at least 2 °C. The replacement of the controller box with the lightweight controller capsule saves space. Like the LPRTs, the CLRTs also include a sapphire lightpipe enclosed in a concentric sapphire sheath (see Fig. 1c). In addition, CLRTs are accompanied by software for reading and acquiring the temperature and signal data and contain a small electronics controller cylinder which houses the optics and electronics. Disadvantages of CLRTs include the shorter lengths and the inflexibility of the cables.

## 3. Calibration of LPRTs

### 3.1 Standard Reference Blackbody Source

**Fig. 2:**
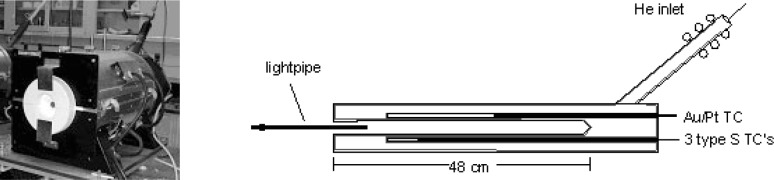
Left: photograph of NIST sodium heat pipe blackbody. Right: schematic of sodium heat pipe blackbody with lightpipe properly inserted.

At NIST, LPRTs, STRTs, CLRTs, and other types of RTs, are routinely calibrated against a sodium heat pipe blackbody (Na-HPBB). The main Inconel^1^ cavity of the Na-HPBB shown in Fig. 2 is 25 mm in diameter and 48 cm in length, whereas its aperture opening is 22 mm in diameter. Surrounding the cylindrical cavity is a 90 mm diameter tube, which contains the sodium liquid and vapor. A condensing tube at the rear of the black-body allows the metal vapor to liquefy back into the tube and at the same time serves as the conduit by which the tube is pressurized with helium. A Monte Carlo model [32] was used to estimate the cavity emissivity as 0.99992 ± 0.00003 for the wavelength range from 1 µm to 5 µm with an Inconel surface emissivity of 0.85. The Na-HPBB temperature, which is measured by a gold-platinum (Au/Pt) thermocouple (TC), is computer-controlled by regulating the pressure of the helium. Three type S thermocouples monitor the temperature in three zones along the cavity. Using the Au/Pt TC links the Na-HPBB temperature to the ITS-90. Further details of the Na-HPBB are given in [32].

Uncertainties (*k* = 1 [67] is implied throughout this paper unless otherwise specified) for the Na-HPBB are provided in Table 1. The dominant component in the Na-HPBB temperature uncertainty is the blackbody radial uniformity as viewed and measured by a RT in front of the Na-HPBB. The blackbody stability and the uncertainty in the calibration of the Au-Pt TC are small in comparison with the dominant uncertainty.

### 3.2 Calibration Procedures for Spot-Type Radiation Thermometers

The procedures for preparing and operating the Na-HPBB are the same, regardless of the RT type (LPRT, STRT, or CLRT). Safety checks are performed, prior to turning on the power for the Na-HPBB, to ensure adequate water cooling, sufficient helium, and the proper electrical connections. Next, the heater power is turned on, and the helium pressure adjusted to raise the black-body temperature to a desired set-point temperature.

**Fig. 3:**
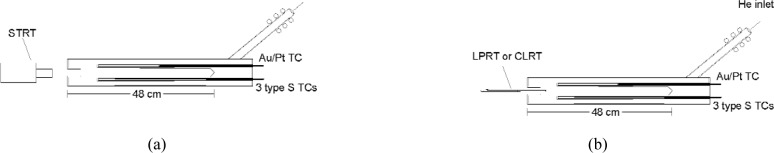
(a) Calibration of STRT. (b) Calibration of LPRT or CLRT.

Before a STRT is calibrated, it is visually inspected for scratches or dents, breaks in cables, excessive dirt, or other obvious problems. If serious damage is discovered, it is immediately reported to the owner of the STRT. Otherwise, the lens, eyepiece, and the filters are cleaned with lens tissue paper and ethyl alcohol to remove fingerprints and sprayed with air to remove dust. Next, the front surface of the lens is positioned to a specified distance from the blackbody aperture, as shown in Fig. 3a. A level situated on top of the STRT is used to ensure that the STRT is horizontal. The yaw and pitch of the STRT are tweaked to be assured that the optical axis of the STRT is coincident with the geometrical center of the Na-HPBB. The centering process is usually done first roughly by using eyes to sight on the center and then more precisely by using a computer program to search for the radiometric center. Then, the STRT focus knob, if existent, is adjusted to find the maximum signal or temperature. After the Na-HPBB is stabilized to within 30 mK, three STRT measurements are acquired in one-minute intervals and are averaged. With each STRT reading, a measurement of the Au/Pt TC is recorded. The three TC readings are also averaged, and the difference Δ*T*, the TC temperature minus the average STRT temperature, is recorded as the offset temperature. To calculate the corrected temperature, the offset temperature is added to future RT readings.

### 3.3 Calibration Procedures for Lightpipe Radiation Thermometers and Cableless Lightpipe Radiation Thermometers

On a routine basis, the LPRTs and CLRTs are calibrated against the Na-HPBB before and after measurements. The LPs undergoing calibration are visually inspected for dirt, and their tips cleaned with a tissue wiper or a cotton swab saturated with ethyl alcohol. After the Na-HPBB comes to a stable temperature and does not vary more than 30 mK, the LP is rapidly inserted into the Na-HPBB in Fig. 3b, measurements of the LPRT or CLRT indicated temperature are recorded, and the LP is removed before it heats up by more than 0.2 °C. The measurements usually take about 5 s to 10 s and are referred to as a cold calibration. Before and after their use in our test bed experimental studies, a set of LPs is calibrated in this way, and the temperature of the Au/Pt TC is recorded. For each LP, three measurements are averaged and the difference, the average temperature minus the TC temperature, is recorded as the offset temperature. The temperature of the Na-HPBB is then increased to the next temperature, and the whole procedure repeated.

When the LPs are visually contaminated (with carbon deposits or other contaminants), or when the LP response in RTP measurements or calibration changes by more than 2 °C, the LPs are cleaned using a flame cleaning procedure. With the outer sheath removed, the LP is first wiped with acetone and ethanol and then heated with an oxygen-methane flame to remove any contamination. Care is exercised to heat the LP slowly and uniformly to avoid damage.

After the LPs have been cleaned through the flaming process, or after the LPs are returned from the factory calibration, the sensor factor settings need to be adjusted. The adjustment is performed by changing the LP sensor factor setting until the LP indicated reading is within 0.02 °C of the Au/Pt TC reading for the Na-HPBB at the highest calibration temperature, 900 °C. A few LP temperature readings are obtained for establishing repeatability. The LP sensor factor setting is recorded and stored for the remainder of the calibration procedure and for future LP measurements. It should be noted that after the LP is cleaned and calibrated, it remain attached to the LPRT until the next flame cleaning is required.

### 3.4 Calibration and Measurement Uncertainties

Table 2 displays the uncertainties for calibrating LPRTs. The principal uncertainty is the uncertainty in the Na-HPBB temperature determination from Table 1. In comparison with this uncertainty, the other uncertainties in the LPRT, stray radiation effects, and the Na-HPBB emissivity, are negligible. Since it is very difficult to replicate the exact position and cable looping of the LPRTs in the calibration mode, the uncertainty due to the handling and positioning of the LPRTs is estimated to be about 2.0 °C. Although the uncertainty in the NIST calibration of LPRTs is only 0.31 °C, the total estimated uncertainty taking into account the handling uncertainty, is about 2.02 °C.

Uncertainties for calibration of STRTs are shown in Table 3. The dominant uncertainty factors are the Na-HPBB temperature uncertainty and the STRT resolution. The other uncertainties in the STRT, stray radiation effects, and the Na-HPBB emissivity, are small. The total estimated uncertainty in a typical calibration of a STRT using the Na-HPBB is about 1.05 °C and is limited by the resolution of the STRT. Improving the STRT resolution will decrease the total uncertainty.

Table 4 shows the uncertainties for calibrating a CLRT using the Na-HPBB. The uncertainties for the CLRT calibration are exactly the same as the ones for the LPRT calibration with one exception. That is, the large uncertainty component due to the handling of the cables is eliminated. The total estimated uncertainty in a typical calibration of a CLRT using the Na-HPBB is about 0.31 °C.

In Tables 2, 3, and 4, the uncertainties of 0.31 °C, 1.05 °C, and 0.31 °C, respectively, represent the uncertainty of the RT calibration. Other factors, which will increase the uncertainty, need to be considered when approximating the total uncertainty in using the RT for temperature measurement.

### 3.5 LPRT Stability

**Fig. 4:**
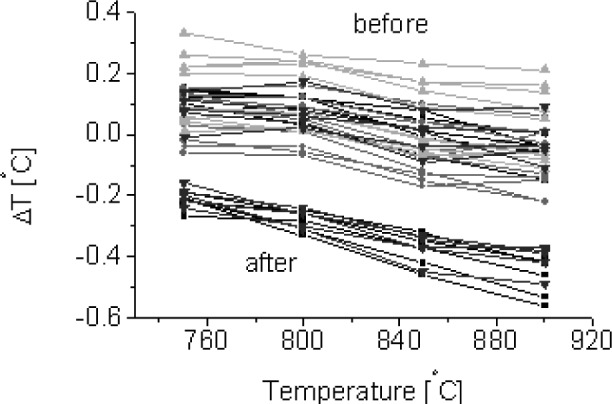
LPRT calibration in *cold* mode, including those before and after cleaning of the LPRT.

After use in the RTP test bed for about a month, the LPRTs are calibrated again to check for variability during use. In Fig. 4, typical calibrations of four LPs are shown for a period of 1 year, including those before and after cleaning of the LPRT. Variations during this period of time were less than 1 °C for all four LPs.

### 3.6 LPRT Application Issues: Factory vs NIST Calibrations and Hot vs Cold Measurements

Applying calibrated LPRTs in an industrial or process application is more complicated and requires more analysis for determination of temperature, as well as establishing uncertainty limits and traceability. In this section, we present two issues associated with using LPRTs in applications outside of a well-controlled laboratory environment. Differentiation is made between hot and cold calibration for LPRTs. Our recommendation is to calibrate in the same fashion as the application.

**Fig. 5:**
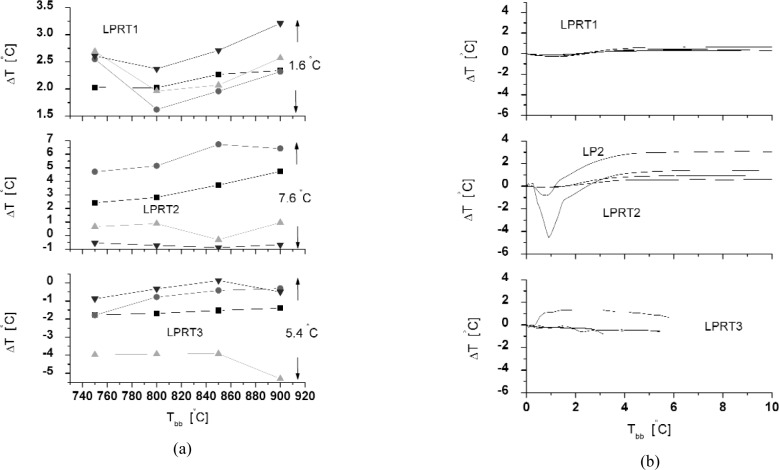
(a) Factory vs NIST *hot* calibrations. (b) *Hot* vs *cold* calibrations. (Values for four different LPs are shown in each graph.)

Three LPs from different vendors, using the factory-set sensor factors, were calibrated using the Na-HPBB as soon as they were received at NIST. The differences between the LPRT indicated temperatures in the factory hot-mode calibrations and the actual temperatures measured with the Au/Pt TCs in the Na-HPBB are shown in Fig. 5a. From the results for three LPRT systems (LPRT1, LPRT2, LPRT3), each system consisting of four LPs, the variations among the four LPs in LPRT2 can be as high as 7.6 °C, while those for LPRT1 are as low as 1.6 °C. Thus, without measuring this difference shown in Fig. 5a, the user will not know the magnitude of the uncertainty in using a set of LPs.

In the Na-HPBB, time histories of all of the LPs at 850 °C are shown in Fig. 5b. After the LP is inserted into the Na-HPBB for a few seconds, the LPRT indicated temperature initially remained fairly constant, dipped, and then rose above the initial temperature to a steady temperature. *Cold* calibrations were performed in the initial period when the temperature was still constant, while *hot* calibrations were performed after the Na-HPBB was stable during the second temperature rise. Although most LPRTs behaved in this manner, other patterns have been observed. However, in general, there was a constant plateau during the first 30 s or 1 min (*cold*) and again after 5 min (*hot*) from insertion. Significant differences exist between *hot* and *cold* LP calibrations in Fig. 5b of up to 2.5 K for LPRT2, while only modest differences of up to 0.7 K for LPRT1 were exhibited. These findings correlated very well with visual and transmission measurements to be discussed in Sec. 4.4.

When performing *hot* calibrations, the following issues can influence the accuracy of calibration and measurement errors in RTP tools. First, the LP can become contaminated by ceramic particles or impurities in the blackbody, especially if the LP is left in the blackbody for more than several minutes. Second, the inserted LP disturbs the temperature distribution and thus changes the relationship between the spectral radiance sensed by the LPRT and the reference temperature of the blackbody. Quantifying this perturbation requires further experiments or heat-transfer modeling. Third, the uncertainty of the measured blackbody temperature needs to be determined. If TCs are used for the reference temperature, an uncertainty analysis needs to be performed so that the blackbody temperature uncertainty can be related to the TC uncertainty, as well as, to other features of the blackbody, such as temperature non-uniformities and non-unity emissivity. If other blackbody sources, such as fixed points, are used, the uniformity and accuracy of these blackbodies need to be characterized. Fourth, the frequency of recalibration of the NIST-traceable TC providing the reference blackbody temperature needs to be established to avoid significant changes with use. Fifth, leakage can occur through the lateral side of the LP as a consequence of surface roughness, which can be worsened by the presence of gradients in the refractive index along the LP. Leakage can be minimized by using *cold* calibrations, or by using a cold radiation shield such as the cold sleeve. The first two issues above are unique to *hot* calibrations, while the last issue can be minimized using *cold* calibrations. The other two issues are common to both *hot* and *cold* calibrations.

To make accurate LP temperature measurements, it is necessary to understand the accuracy of factory calibrations, the difference between *hot* and *cold* calibrations, and the importance of visualization and measurement techniques in defect detection for LPs. An understanding of these practical principles will be helpful not only in making more accurate LP temperature measurements but also in choosing a quality LP and in developing an improved LP calibration system.

### 3.7 Calibration Services

Calibration services are available at NIST for RTs from 15 °C to 2700 °C using various blackbodies. For the temperature range from 700 °C to 900 °C, the Na-HPBB is used as the standard blackbody source. Calibration reports are issued giving the thermodynamic temperature of the reference blackbody (BB) vs the radiation thermometer (RT) display reading, output current, or output voltage. Users can order one of two calibration tests from the NIST Calibration Services manual [68] or from the NIST Calibration Services website [69]. The first is service ID number 35084C for a Radiance Temperature Standard, Radiation Thermometer (700 °C to 900 °C, three points). This calibration is a set-fee measurement at any three temperatures between 700 °C and 900 °C. The second is service ID number 35080S for Special Tests of Radiation Thermometers (15 °C to 900 °C). Since this special allows the user to customize the measurement set, the price is uniquely determined for each situation. Any questions or requests for quotes may be addressed to the author at benjamin.tsai@nist.gov.

## 4. Characterization

Throughout this section, data from LPRTs for three different vendors are discussed. The term LPRT refers to the measurement system including the controller unit, the quartz fiber-optic cable, and the sapphire LP. Hereafter, the three LPRTs will be referred to as simply LPRT1, LPRT2, and LPRT3. Each LPRT consists of four channels, which are connected to four sapphire LPs (LP1, LP2, LP3, and LP4). Since there are twelve LPs in total, each LP will be identified by LPRT and LP numbers (e.g., LPRT2-LP3). The term LP only refers to the sapphire rod, which is the part of the LPRT aimed directly at the target. The LPs are of varying lengths, but they are 2 mm in diameter and are typically surrounded by a 4 mm outer diameter sapphire sheath. In normal operation for measuring the spectral radiance temperature, the LPs are connected to a 1 mm diameter quartz fiber-optic cable. The exposed length of sapphire sheath is about 9 cm. For more details, see [32].

### 4.1 Point Spread Response

**Fig. 6:**
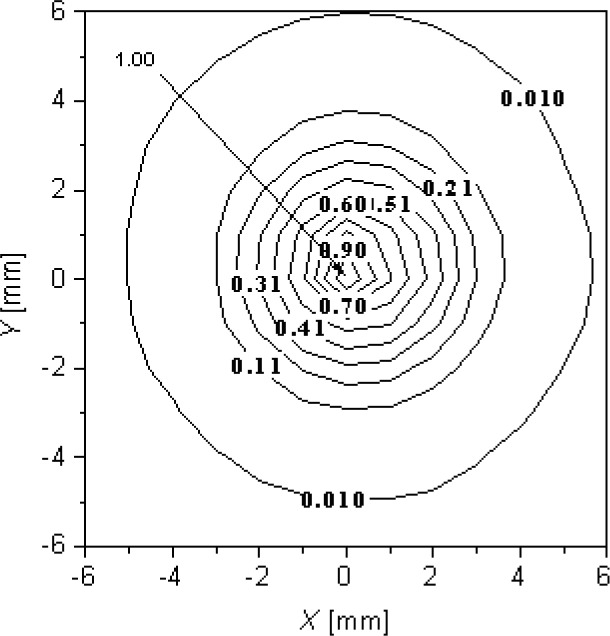
Target size determination from a small lamp bulb 12 mm from front of LPRT1-LP1.

The effective target area on a silicon wafer viewed by the LPRT was determined in the point spread response (PSR) facility. Attached to a precision x–y stage, the LP was translated under computer control in a vertical plane to measure the radiation emanating from a small stationary lamp bulb (about 2 mm). The normal distance from the source to the vertical LP plane was carefully set to coincide with the corresponding wafer-to-LP tip gap separation distances in the NIST test bed. From the resulting intensity distribution of the measured radiation, the wafer spot was chosen to be the area that enclosed intensities greater than 1 % of the maximum intensity. This technique was repeated for different lamp-to-LP tip gap separations. The contours of the PSR measurement in Fig. 6 indicate the fraction (with 1.00 being equal to 100 %) of the maximum intensity measured at the origin of the vertical plane in which the LP is translated. This origin is located at the same *X* and *Y* location as the lamp bulb. Figure 6 shows that the target size for a gap separation of 12 mm is about 12 mm in diameter for LPRT1-LP1. This spot-size information is useful in the modeling of the effective emissivity, determination of the corrected LPRT spectral radiance temperature, and setting up the LPRT for measurements.

### 4.2 Absolute Spectral Response

A spectral characterization of the LPRTs was performed using the Spectral Comparator Facility (SCF) [70], in which the LP fixed on a linear translation stage was aligned with the center of a monochromator slit and was used to collect the output of a spectrally filtered beam from a quartz-halogen source through the monochromator. This measurement was compared with that using a standard trap detector, calibrated previously against the NIST Primary Optical Watt Radiometer (POWR) [71], the successor of the NIST High Accuracy Cryogenic Radiometer (HACR) [71].

**Fig. 7:**
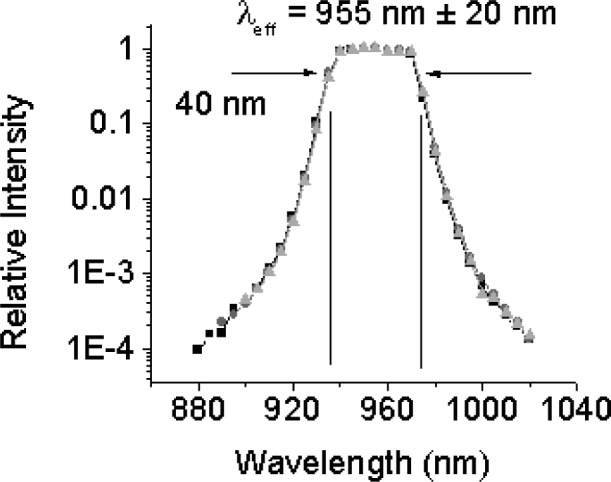
Relative spectral response for LPRT1-LP1, LPRT3-LP3, and LPRT3-LP3, using the SCF.

The relative response curves, or the absolute spectral response curves normalized to unity, for three LPs (LPRT1-LP1, LPRT3-LP3, LPRT3-LP4) obtained using the SCF are depicted in Fig. 7 and are very similar. Based on the full width at half maximum, the peak for all three LPs is centered about an effective wavelength of about 955 nm with a bandwidth of 40 nm. The effective wavelength is critical in the determination of the surface temperature from the LPRT spectral radiance temperature by using the temperature measurement equation. In addition, the effective wavelength and the spectral bandwidth are useful in the estimation of the temperature uncertainty [39]. Outside of the 40 nm bandwidth, the relative response quickly decreases four orders of magnitude outside of a bandwidth of about 140 nm. This information can also aid in the uncertainty analysis as well as in the quality control of LPs. The similarity of all three curves in Fig. 7 reveals the consistency and quality of these LPs and their filters, which come from two different vendors.

### 4.3 Temporal Response

**Fig. 8:**
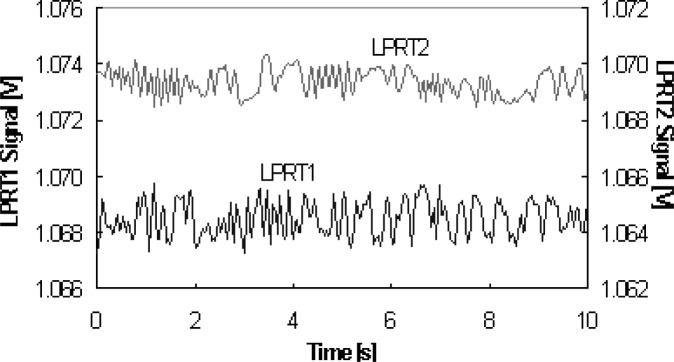
Typical temporal stability for two LPs under stable temperature conditions.

In Figure 8, the temporal stability for a period of 10 min is shown for two LPs, LPRT1 and LPRT2. The results were obtained by irradiation from a helium-neon (HeNe) laser into the LP while it was in an integrating sphere. The resulting variations at room temperature for LPRT1 and LPRT2 were about ±0.06 % and ±0.04 %, respectively. This corresponds to a temperature standard uncertainty at 1000 °C of 0.064 °C and 0.043 °C, respectively.

### 4.4 Optical Characterization of Lightpipes

A measure of the LP quality is the radiation scattering from the lateral surface along the length of the LP. For an ideal LP, the scattering effect will be zero. However, in reality, defects in the manufacturing process can lead to surface imperfections that can cause loss of radiation from the lateral surface. To determine whether such defects are contributing to differences in calibration, two specific studies were conducted for LPRT1-LP2 and LPRT2-LP2. Both studies were made by passing a HeNe laser beam along the LP and by observing the circumference for irregular patterns. The first study qualitatively showed a relatively large number of bright spots for LPRT2-LP2. This visual study emphasized the need for a more quantitative experiment to determine the radiation loss from the lateral surface due to scattering. An experiment using an integrating sphere and a silicon detector to measure the transmitted and scattered signals constituted the second study that was conducted on the same two LPs used in the first study.

#### 4.4.1 Visual Inspection of Lateral Surfaces

**Fig. 9:**
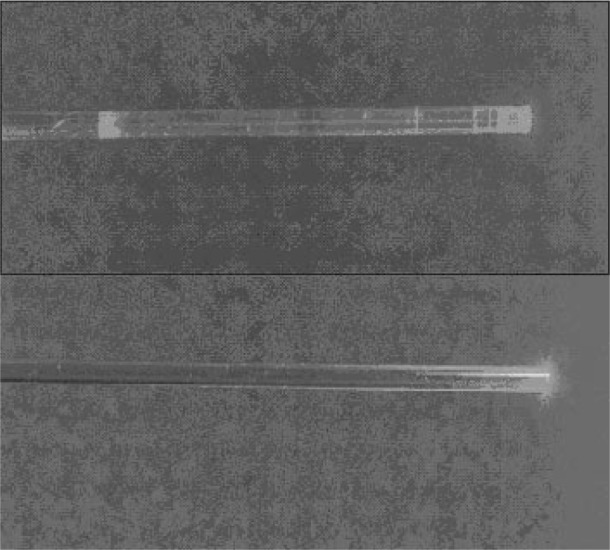
Comparison of a lightpipe with excessive scattering (top, LPRT2-LP2) with a good lightpipe (bottom, LPRT1-LP2) using HeNe laser.

A HeNe laser beam (0.95 mW at 637 nm) was used for irradiating the end of the LP in both studies. A 1 mm diameter quartz fiber-optic cable transmitted the beam from the laser to the 2 mm diameter sapphire LP. To compare the whole length of sapphire rod from both LPs, the LPs in Fig. 9 were photographed while the HeNe laser illuminated the LP. The top part of the figure (LPRT2-LP2) reveals many defect locations where significant scattering can take place, while the bottom (LPRT1-LP2) reveals a more perfect sapphire crystal structure, resulting in less scattering. Figure 9 clearly shows the utility of a simple visualization technique, such as the one used in this study, to detect scattering defects in LPs before calibration.

#### 4.4.2 Quantifying Lateral Scattering

For the second study, an integrating sphere, about 18 cm in diameter, fitted with a silicon detector was used to measure the radiance of the laser beam with and without the LP inserted. The laser beam entering the sphere was distributed uniformly on the inner surface of the sphere by multiple reflections. The output of the silicon detector was proportional to the laser power incident on the sphere surface. The low-level current signal from the silicon detector was amplified by a current amplifier, and the output voltage measured by a digital voltmeter. Data recording by the voltmeter was performed by a computer.

**Fig. 10:**
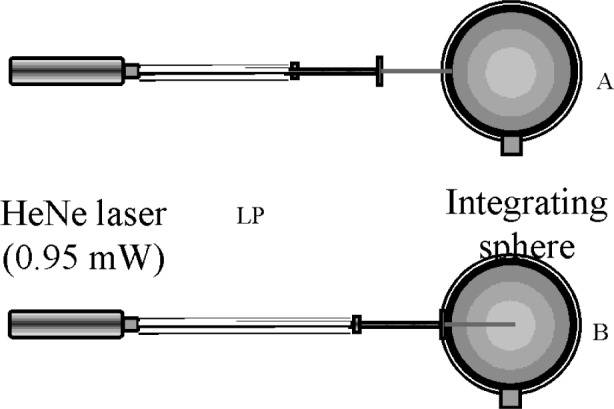
LP positions for measuring transmitted and scattered signals.

The two positions of the LP in the integrating sphere, A and B, in the second study are shown in Fig. 10. In Position A, the tip of the LP was positioned in the plane of the integrating sphere aperture. In this position, only the portion of the radiation transmitted through the length of the LP was distributed onto the integrating sphere surface. In Position B, the LP was inserted inside the sphere cavity with the exposed portion of the sapphire sheath also inside the cavity. The radiation loss from the sheath was also captured, along with the transmitted beam, by the integrating sphere surface. The difference between the readings in Position A and Position B was a measure of the radiation loss through the lateral surface of the LP. Since this radiation loss was less than 1 % of total power and since intermittent surges in power, lasting several seconds, occurred periodically, the laser measurements were made over a long period of time, and an interval, during which the laser power was stable, was chosen for the analysis. Table 5 shows the final results of the measurements for LPRT1-LP2 and LPRT2-LP2 before and after cleaning with a flame. For all measurements, the dark signal, which was the measured signal taken without the laser and with the integrating sphere aperture covered, was less than 0.001 mV.

LPRT2-LP2 showed nearly twice as much radiation loss through the lateral side as LPRT1-LP2. This correlated with LPRT2-LP2 having more defects. Hence, the present study suggests that the integrating sphere method can be used to identify and qualify LPs suitable for use in RTP chambers to achieve the desired accuracy. Such qualified LPs can then be calibrated for spectral radiance temperature using primary-standard blackbodies. The method can be improved by using a more stable laser.

#### 4.4.3 Correlation of Optical Characterization Results With Hot/Cold Calibration Results

In Sec. 3.7, LPRT2 exhibited differences between hot and cold LP calibrations of up to 2.5 K, while LPRT1 only showed differences of up to 0.7 K. The optical characterization study shows that visual effects could be correlated with quantitative measurements. Visual defects as well as the ratios of the scattered signal to the transmitted signal have direct relationships with the amount of thermal leakage as measured by the net temperature rise in 10 min. The visual defects and transmission measurements both have a strong correlation with the difference between *hot* and *cold* LP calibrations. LPRT2-LP2 exhibited a large difference of 2.5 K in Fig. 5b and a large slope in Fig. 5a. It also showed the most visual defects from the optical characterization. On the other hand, LPRT1-LP2 showed only a difference of 0.5 K in Fig. 5b and a slight slope in Fig. 5a. This LP was relatively clear of visual defects from the optical characterization. In order to make accurate LP temperature measurements, it is necessary to understand the accuracy of factory calibrations, the difference between *hot* and *cold* calibrations, and the importance of visualization and measurement techniques in defect detection for LPs. Both types of experiments are crucial in detecting LPs that may exhibit significant scattering.

### 4.5 Stray Light Effects on the LPRT Indicated Temperature

**Fig. 11:**
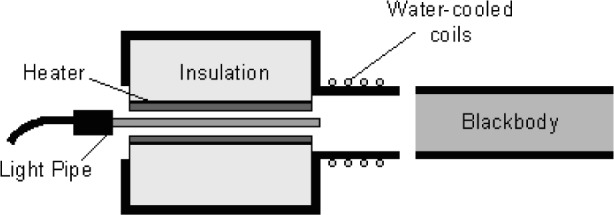
Schematic of furnace experiment.

**Fig. 12:**
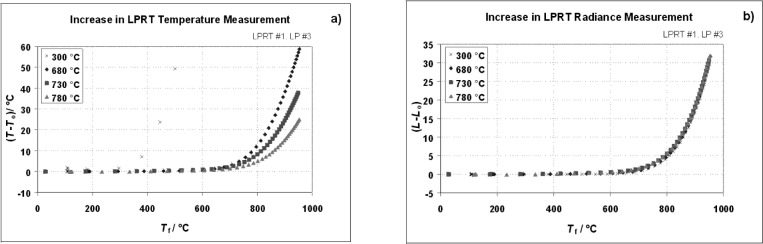
Graphs as function of furnace temperature for a) temperature increases and b) radiance increases.

Two experiments were performed with the Na-HPBB to study the stray light effects on the LPRT indicated temperature. The first examined the influence of a hot environment on the indicated LPRT temperature by surrounding the LP lateral surface in the furnace of Fig. 11 and heating it while the LP was aimed at a constant radiance source, the Na-HPBB. The increases in the LP indicated temperature and the resulting radiance were plotted as a function of the furnace temperature for four different initial LP indicated temperatures *T*_o_: 300 °C, 680 °C, 730 °C, and 780 °C. Figure 12a shows that for LPRT2-LP3 the temperature increase was largest at the highest furnace temperature (950 °C), but it was always less than 4 °C when the furnace temperature was at or below the Na-HPBB temperature. However, when the temperature differences are converted to radiance differences, the radiance increases in Fig. 12b are independent of the Na-HPBB temperature. Thus, extraneous radiation can reach the LP through its lateral surface, but the radiance increase is only dependent on the temperature of the LP surroundings.

**Fig. 13:**
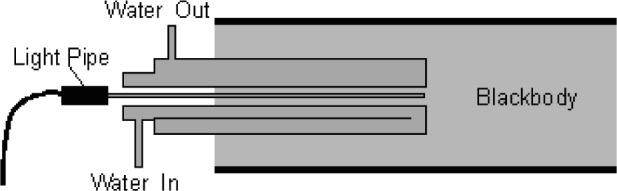
Schematic of cold sleeve experiment.

**Fig. 14:**
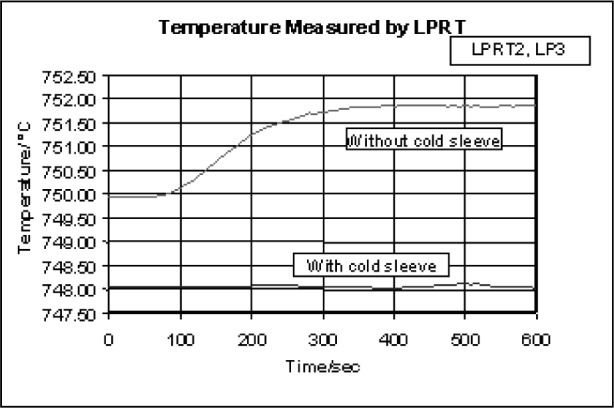
Temperature indicated by LPRT1-LP3 in the 750 °C Na-HPBB as a function of time.

The second experiment, illustrated in Fig. 13, examined the influence of the Na-HPBB environment on the temperature indicated by an LPRT by taking measurements with and without a water-cooled, stainless-steel sleeve, which maintained the LP temperature below 100 °C and blocked radiation from entering its sides. The temperature history for LPRT2-LP3 in Fig. 14 shows that the indicated temperature without the cold sleeve drifted higher by 2 °C over 400 s before becoming steady, and the initial indicated temperature was higher by 2 °C before the LP was significantly heated. With the cold sleeve, the LPRT temperature did not drift. The results at *t* = 0 quantify the blackbody calibration error due to light scatter from irradiation of the unsleeved LP from the sides. The drift in indicated temperature of the unsleeved LP over the first 400 s in the Na-HPBB suggested that additional radiation was emitted from the LP after it reached a sufficiently high temperature. Perhaps this emission was due to impurities in the LP sapphire crystal. This result shows that some LPs are less susceptible to extraneous radiation than others and suggests that better manufacturing techniques or materials for LPs can minimize calibration errors due to these effects [33,79].

### 4.6 Discussions and Recommendations

Based upon our LPRT calibration and characterization experiences at NIST, we offer the following recommendations for users of LPRTs in calibration or measurement applications:

#### 1. Visually inspect the LP first

Before any measurement is performed, the LP should be inspected for defects. A visual inspection can detect macroscopic chips and nicks. For more detail, the simple laser techniques and the more complex methods using integrating spheres or hot furnaces can assist in qualifying high-quality LPs.

#### 2. Understand the factory calibration

When factory calibration data is available, the user should always try to verify the data when possible. The user should find out whether the factory calibrations were performed using the *hot* or *cold* calibration mode. The LP calibrations should then be checked using blackbody or other radiance sources for determining the spectral radiance temperature. Alternatively, the user could implement an *in situ* LPRT calibration method, such as the NIST thin-film TC test wafer [21], to determine the surface tem perature. If the results match the factory data, then the user can proceed with the LP measurements. Otherwise, the user should determine which set of calibrations to use by selecting the calibration conditions that best fit the measurement conditions. If the user is confident of the checking process and conditions, then the user calibration should be employed in LP measurements. It is good practice to check the factory calibrations against available blackbodies or other sources of low radiance temperature uncertainty.

#### 3. Characterize the LPRT

The LPRTs should be characterized spectrally, spatially, and temporally with available resources. The LPs should be measured in the appropriate wavelength region to check the peak effective wavelength and the narrowness of the bandwidth for single-wavelength temperature measurements. Some check of the point spread response should be performed to estimate the field of view of the LPs to assist in analysis and modeling. The drift of the LPs for an appropriate period of time should be determined for the LPs at several temperatures to help assess the measurement uncertainties.

#### 4. Minimize lateral scattering

Wherever possible, a method to minimize lateral scattering through the LP, such as a cold sleeve, should be used for *cold* calibrations. This will ensure that extraneous radiation is eliminated in LP calibrations and that the LP remains at a cold temperature.

#### 5. Calibrate the LPRT as it will be used

The cardinal rule of LPRT calibrations is to calibrate in the same manner or as close to the same way in which it will be used. If the LP tip will be used in a cooled environment, such as the NIST RTP test bed, then the LP tip should be calibrated in the *cold* mode. If the LP tip will operate in a hot surroundings, then the LP tip should calibrated in the *hot* mode. It should be noted that the LP tip may be the only hot part in the *cold* mode. An understanding of the LPRT surroundings will aid in making better LP calibrations.

#### 6. Calibrate the LPRT using blackbodies with traceable calibrations

The LPRT should be calibrated using blackbodies traceable to the SI unit of temperature. For highest accuracy, the spectral radiance of the blackbodies should be traceable to blackbodies at NIST or another national measurement institute (NMI). Alternatively, the temperature of the blackbodies could be traceable to the blackbody TC, which is traceable to the SI unit through an NMI.

#### 7. Calibrate before and after use

Immediately before and after LP use, the LPs should be calibrated to check for any systematic drift or change. If there is any significant change in calibration, the LPs should be inspected again for any damage or contamination during measurement, moving, or shipping of the LPs.

These practical principles have been formed from our experience with calibrations and measurements of LPs from several vendors. Following these guidelines wherever possible can ensure highly accurate LP calibrations and temperature measurements on the ITS-90.

## 5. Comparison With Thin-Film Thermocouple Wafers

### 5.1 Experimental Procedure and Equipment

**Fig. 15:**
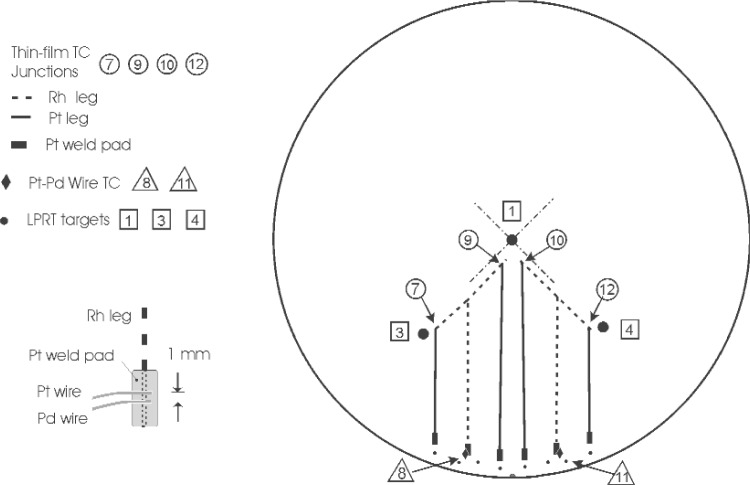
Schematic of the wafer layout with thin-film thermocouples, wire thermocouples, and lightpipe radiation thermometer targets.

The silicon calibration wafer in Fig. 15 was instrumented with the new wire Pt/Pd TCs [73,74] and the new Rh/Pt TFTCs [19]. The region at the center of the wafer in close proximity to the TC junctions is the primary target for sighting by the LPRT. The thin films were sputter deposited on oxidized silicon wafers using physical masks for the 0.5 mm thick metal films of 99.99 % Pt and 99.95 % Rh, and the films were bonded to the SiO_2_ with sputter-deposited Ti. This procedure is described in more detail by Kreider and DiMeo [18]. The thin-film pattern included welding pads 10 mm from the edge of the wafer for the 0.25 mm diameter Pt/Pd TC wires. The uncertainty in temperature measurement of the thin-film thermocouple junction with this design is 0.3 °C with a temperature difference of up to 10 °C from the center to the edge of the wafer.

**Fig. 16:**
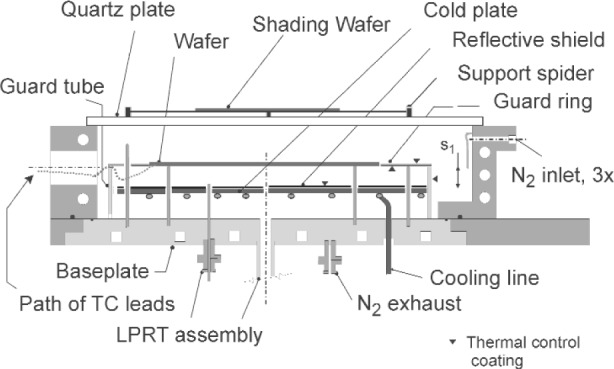
The NIST RTP test bed.

The LPRTs were first calibrated using the Na-HPBB according to the procedures described in Sec. 3. Then the LPRTs were carefully transported without disconnecting cables and installed in the RTP test bed in Fig. 16. A detailed description of the test bed can be found in Ref. [36]. Comparisons between the temperatures measured by the TCs and the LPRTs in the RTP test bed were performed after reaching steady state while a constant heating power was applied.

### 5.2 *In-Situ* Calibration of LPRTs

**Fig. 17:**
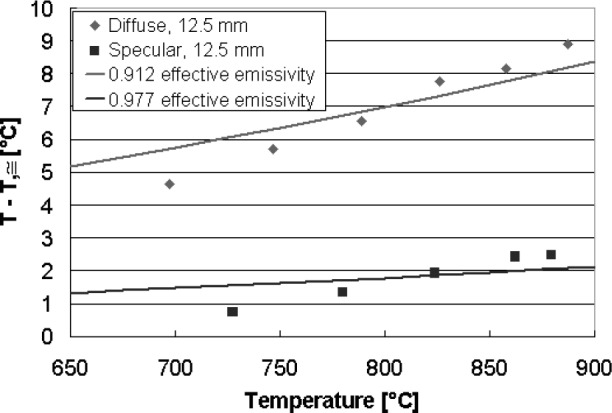
Values of *T*_tc_ − *T_λ_* near wafer center.

Figure 17 shows a comparison between the temperatures measured by the thin-film thermocouple wafer (*T*_tc_) and those measured by the LPRT (*T_λ_*) for a diffuse and a specular shield with a wafer/shield spacing of 12.5 mm. The values of *T*_tc_ − *T_λ_* for the specular shield shown in squares are 1.8 °C ± 0.7 °C, while the values for the diffuse shield are larger. This is expected, because the reflectance of the specular gold shield (*ρ* = 0.993) is higher than that of the diffuse gold shield (*ρ* = 0.799), implying a larger *ε*_eff_ for the specular shield. For both shields, the temperature accuracy of the LPRT will be improved by *in situ* calibration. By curve fitting, the *ε*_eff_ for the specular and diffuse shields were estimated to be 0.98 and 0.91, respectively.

**Fig. 18:**
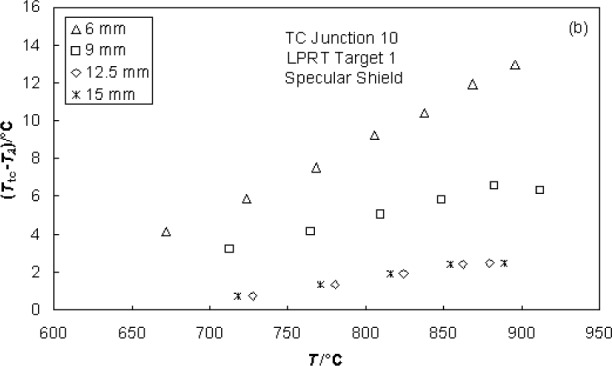
Values of *T*_tc_ − *T_λ_* near wafer center for four different wafer/shield spacings.

Figure 18 shows the effects on *T*_tc_ − *T_λ_* of changing the wafer/shield spacing. For this plot, the specular shield was used. While the results for spacings of 12.5 mm and 15.5 mm are identical to within the resolution of the measurements, the values for *T*_tc_ − *T_λ_* increase as the spacing is decreased from 12.5 mm to 6 mm. This effect can be explained by the optical perturbation on *ε*_eff_ of the LPRT target area caused by the presence of the LP, which has a much smaller reflectance (*ρ* = 0.075) than the shield. Because the LP occupies a large solid angle of the field-of-view as seen from a point on the wafer when the wafer is close to the shield, an *in situ* calibration should be performed with the same spacing as in the application.

### 5.3 Uncertainties

According to Table 6, the dominant uncertainty of 2.0 °C for the *in situ* LPRT calibration arises from the physical separation of 1.4 cm between the TFTC junctions and the center of the LPRT target and is based on the assumption of a uniform temperature gradient of 10 °C in this separation [22,25]. However, no correction for temperature gradients was ever applied to the calibration measurements. Other measurement uncertainties include thermocouple calibration uncertainties, from temperature fluctuations and long-term temperature drift of the wafer while in steady state, LPRT calibration uncertainties, and instrument uncertainties for temperature measurement with the thermocouples and LPRTs. The standard uncertainty for the *in situ* LPRT calibration is 2.3 °C.

## 6. Effective Emissivity Models

**Fig. 19:**
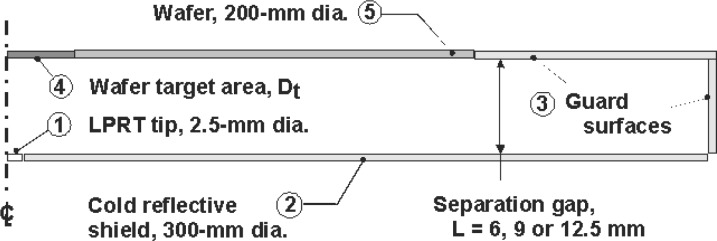
Cross-section schematic of the classical diffuse/specular enclosure.

To establish the uncertainty of LPRT measurements for wafer temperature, it was necessary to develop models for estimating the effective emissivity of the wafer that include effects due to wafer emissivity, shield reflectivity, lightpipe (LP) sensing tip area, and guard surface geometry and their radiative properties. Comparison of the TC and model-corrected LPRT temperature measurements are presented and the uncertainties of the LPRT calibration are described. Measurements of the room-temperature, directional-hemispherical reflectance for the RTP chamber reflective shields and the silicon wafer in Fig. 19 were obtained using the NIST Spectral Tri-function Automated Reference Reflectometer (STARR) [75] and were used to determine shield emissivities. The room temperature spectral reflectance at *λ* = 0.955 µm for the silicon wafers used in our study was measured as 0.686, which is in close agreement with the database [76] value at 30 °C of 0.680 from which the high temperature emissivity values were estimated.

### 6.1 Wafer-Chamber Arrangement: the Radiation Enclosure

The simplest model for predicting the wafer effective emissivity represents the wafer-shield as a two-surface (infinite-parallel planes) enclosure. If the separation gap between the wafer-shield is very small, and there are no appreciable temperature gradients across the wafer, this model is appropriate. However, this model cannot account for the effects caused by the presence of the cold (nearly black) sensing tip of the LP [16]. Figure 19 illustrates a more realistic model representing the wafer-chamber configuration by five regions: 1) the 4.3 mm diameter LP with 2.5 mm diameter tip, located in the center of the cold reflective shield having a reflectance of 0.0754, 2) the 300 mm diameter *reflective shield*, which is cold and diffuse or specular, 3) the lateral surface *cold wall*, which surrounds the wafer and the reflective shield, and which has a gap separation of *L* and an emissivity of unity, 4) the *LP field-of-view* (target) at the wafer center of a diameter determined by the LP field-of-view and gap separation, and 5) the remaining surface of the 200 mm *silicon wafer*, but not including the LP target. Classical gray, diffuse or specular, enclosure analyses have been performed for this configuration using 24 zones. Since the wafer is assumed isothermal with an emissivity of 0.65 and all other surfaces are assumed cold, the effective emissivity is independent of the wafer temperature and depends only upon the chamber geometry and radiative properties of the enclosure surfaces.

### 6.2 Types of Models

Two models are described for estimating the wafer effective emissivity for the five-zone enclosure shown in Fig. 19. The first model treats the reflective shield as diffuse; that is, for a diffuse-gray enclosure. The second model treats the reflective shield as specular, while the remaining surfaces in the enclosure are diffuse.

#### 6.2.1 Model With the Diffuse Shield

Using the temperature measurement equation with the estimated effective emissivity *ε*_eff_, an estimate of the wafer temperature *T* can be determined from the observed spectral radiance temperature *T_λ_*,

$$\frac{1}{T}=\frac{1}{T_{\lambda }}+\frac{\lambda }{c_{2}}\ln\varepsilon_{\text{eff}},$$ (1)

where *λ* is the operating wavelength of the radiation thermometer and *c*_2_ is the second radiation constant, 14,387.752 µm × K. For the diffuse shield, the enclosure model is developed using the classical, radiosity method [39,77] in which a radiation energy balance is written for each surface (zone) *A_i_* of the *N*-zone enclosure of the form,

$$\sum_{j=1}^{N}\left[\delta_{ij}-\left(1-\varepsilon_{i}\right)F_{i-j}\right]J_{j}=\varepsilon_{i}E_{bi},$$ (2)

where *δ_ij_* is the Kronecker delta function, *E*_b_*_i_* is the blackbody emissivity power, *ε_i_* is the emissivity, *J_i_* is the radiosity, and *F_i_*_−_*_j_* is the diffuse radiation view (or exchange) factor defined as the ratio of radiation leaving an emitting surface *A_i_* to the reflected irradiation that is intercepted by a receiving surface *A_j_*. Using appropriate temperatures, emissivities and the *N*^2^ view factors, the system of *N* equations is solved simultaneously to obtain the radiosities. The radiosity *J_i_* represents the diffuse radiation leaving the surface *A_i_* due to direct emission *and* reflected irradiation resulting from intereflections within the enclosure. The effective emissivity *ε*_eff_ of the target area (t),

$$\varepsilon_{\text{eff}}=\frac{J_{t}}{E_{b,t}}=\frac{J_{t}}{\sigma T_{t}^{4}},$$ (3)

is defined as the ratio of the target radiosity, *J*_t_, to the blackbody emissive power, *E*_b,t_, at the temperature, *T*_t_, of the target area, where the Stefan-Boltzmann constant *σ* is 5.67051 × 10^−8^ W/(m^2^ × K^4^). Since the surfaces are gray, the total and spectral effective emissivities are equal, and this value is used in the temperature measurement equation, Eq. (1), to determine the wafer temperature from the measured spectral radiance temperature.

#### 6.2.2 Model with the Specular Shield

For the specular shield, we have implemented the classical radiation transfer enclosure analysis for specular and diffuse surfaces [77]. In our model, all *N* surfaces of the enclosure emit diffusely, but the *d* diffuse surfaces (*i* = 1, 2, …, *d*; wafer and guard surfaces or zones) reflect diffusely and the (*N* − *d*) specular surfaces (*i* = *d* + 1, *d* + 2, …, *N*; the shield surfaces or zones) reflect specularly. For each diffuse surface, the radiation leaving the surface by direct emission and reflected irradiation is diffuse and is represented by the radiosity, *J_i_*. For each specular surface, the only diffuse radiation leaving the surface is by emission, *ε_i_E*_b_*_i_*; the incident irradiation is specularly reflected. The transport between the surfaces based upon the radiosities *J_i_* and emissive powers *E*_b_*_i_* is determined by the *specular* exchange factor $F_{i-j}^{s}$, defined as the fraction of diffuse radiation leaving *A_i_* that is intercepted by *A_j_* by the direct path *and* by all possible paths involving intermediate specular reflections. Since the shield is planar and is the only specular surface in the enclosure aside from the direct path, there is just one additional path (no multiple specular reflections), thereby simplifying the evaluation of $F_{i-j}^{s}$. The energy balances for each of the diffuse surfaces forms a system of d equations that must be solved to determine the radiosity of the target area,

$$\begin{array}{l} \sum_{j=1}^{N}\left[\delta_{ij}-\left(1-\varepsilon_{i}\right)F_{i-j}^{s}\right]J_{j}=\varepsilon_{i}E_{bi}\\ +\left(1-\varepsilon_{i}\right)\sum_{j=d+1}^{N}\varepsilon_{j}E_{bj}F_{i-j}^{s}. \end{array}$$ (4)

The effective emissivity of the wafer target follows from Eq. (3) as with the diffuse case. Again, Eq. (1) is used to determine the model-corrected LPRT temperature. The radiation balances, Eqs. (2) and (4), for the enclosures with the diffuse and specular shield, respectively, were solved using 24 zones to represent the five regions earlier identified. Each zone is characterized by an area of uniform temperature and emissivity (or reflectivity). The wafer was represented by 10 concentric zones and the shield by 12 concentric zones. The guard ring and the guard tube were each represented by one zone. The resulting system of equations, with one equation for each radiation balance, was solved numerically for the radiosity *J_i_* of each surface by using a standard LU decomposition method [78].

### 6.3 Discussion of Results

**Fig. 20:**
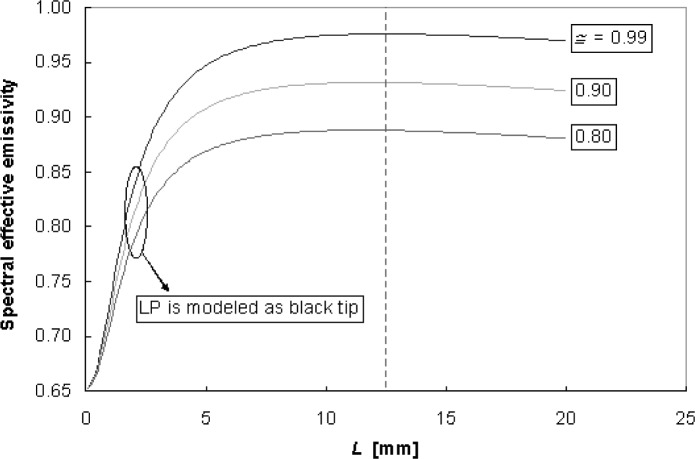
Comparison of wafer effective emissivity for the LP tip with the shield reflectance (0.80 to 0.99) vs a black LP tip.

**Fig. 21:**
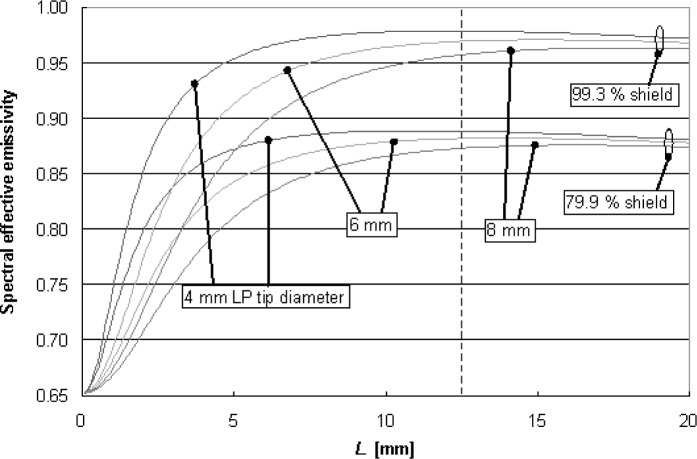
Comparison of wafer effective emissivity predictions for different LP tip diameters.

A parametric study with the two enclosure models showed how the wafer effective emissivity is affected by wafer and shield radiative properties, gap separation, and LP sensing tip area. In Fig. 20, the diffuse-enclosure model shows the effective emissivity as a function of gap separation when the LP sensing tip has the shield reflectance (top set of curves) compared to conditions with a black tip. For the black LP tip condition, as *L* approaches 0 mm, *ε*_eff_ approaches the wafer emissivity. For large gap separations, the influence of the LP tip radiative properties has minimal effect. At *L* = 12.5 mm, the LP tip condition has little influence (less than 0.01 emissivity units or 0.7 K) on the diffuse-model prediction for the effective emissivity. In Fig. 21, the effective emissivity results are based upon the diffuse model, assuming black LP tip areas of different diameters, for shields with reflectance of 99.3 % (specular) and 79.9 % (diffuse). At *L* = 12.5 mm, doubling the LP tip diameter from the 4-mm diameter, representative of the experiment conditions, to 8-mm diameter, reduces the effective emissivity by 0.016 and 0.012 for the 99.3 % shield and 79.9 % shield, respectively. These emissivity changes amount to temperature changes of 0.9 K and 1.2 K, respectively.

**Fig. 22:**
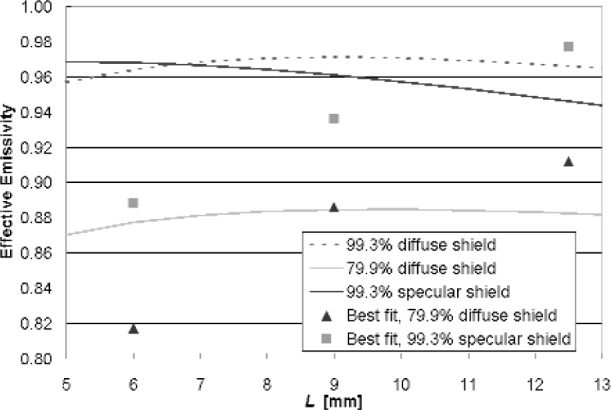
Comparison of TFTC and model-corrected LPRT wafer temperatures (Trad).

The results from the modeling analyses are summarized in Fig. 22 and compared against the experimental effective emissivity values based upon a best fit with the TFTC measurements. The effective emissivity is shown as a function of the gap separation for chamber configurations with diffuse (79.9 % reflectance) and specular (99.3 %) shields. In the limits for very small gap separations, the effective emissivity approaches 0.65, the emissivity of the wafer. At larger gap separations, the effect of shield diffuseness or specularity is less than for smaller gaps. The data points for gap separations of 6 mm, 9 mm, and 12.5 mm, represent the effective emissivity value that provides the best fit between the TFTC measurements and the corrected LPRT measurement of true temperature. Clearly the trends of the data points and the model estimates with gap separation are in poor agreement. We expect the agreement to be poorest at very small gap separations since the interaction between the LP sensing tip and wafer target become increasingly more complicated. Full confidence in the radiation modeling cannot be established until the gap-separation trends and the effects of shield diffuseness are understood.

### 6.4 Uncertainty Analysis

Estimates of the uncertainties from the LPRT and TC measurements are shown in Table 8. A major contributor to the LPRT measurement uncertainty is the effective emissivity uncertainty of about 3.0 °C. The uncertainty due to the difference between the LPRT target and the TFTC junctions is 2.0 °C. Most of the TC total uncertainty is due to the TFTC uncertainty based on the large (10 °C) difference across the 80 mm length of the TFTC. The total LPRT and TC measurement uncertainties are 3.5 °C and 0.3 °C, respectively. From this analysis we conclude that it would be possible to calibrate the LPRT for spectral radiance temperature within 0.5 °C and for actual thermodynamic temperature within 3.5 °C.

## 7. Conclusions and Future Work

We have demonstrated that LPRTs can be calibrated at NIST against a stable blackbody with a standard uncertainty of 0.3 °C, that calibrated LPRTs can be compared against TFTC wafers with an uncertainty of 2.1 °C, and that calibrated LPRTs can be used with model-based algorithms to determine the wafer temperature with an uncertainty of 3.5 °C. A hallmark of the LPRT research at NIST has been the thorough effort of establishing proper calibration and characterization procedures for LPRTs and making critical recommendations for effective LPRT usage protocols. We have stressed the importance of traceability and uncertainty analysis to the RTP community. The utility of models was shown to assist in uncertainty analysis, prediction of properties for chamber design, and achievement of our temperature goals.

For future work, emissivity compensated reflectometer measurements could be a viable alternative for temperature measurements in semiconductor systems. The emittance initiative at NIST has motivated the development of a facility to characterize the emittance of samples as a function of temperature and wavelength. This could prove critical for the semiconductor industry in making more accurate LPRT temperature measurements. The new-generation CLRTs should be investigated as a diagnostic and measurement tool for temperatures up to 900 °C. In particular, CLRTs in the infrared region will be useful for post-exposure bake and other applications in the temperature range from 25 °C to 200 °C.

## Figures and Tables

**Table 1:** Uncertainties in °C for Na-HPBB

Factor	Uncertainty
Na-HPBB radial uniformity	0.29
Na-HPBB length uniformity	0.10
Na-HPBB stability for 1 h	0.03
Au-Pt TC temperature	0.005

Na-HPBB Temperature	0.31

**Table 2:** Typical uncertainties in °C for Na-HPBB calibration of LPRTs

Factor	Uncertainty
LPRT noise	0.01
LPRT short-term drift	0.03
Stray radiation	0.00
Blackbody emissivity	0.03
Na-HPBB temperature	0.31

LPRT calibration	0.31

LP cable handling	2.00

LPRT total	2.02

**Table 3:** Typical uncertainties in °C for Na-HPBB calibration of STRTs

Factor	Uncertainty
STRT resolution	1.00
STRT short-term drift	0.03
Stray radiation	0.00
Blackbody emissivity	0.03
Na-HPBB temperature	0.31

STRT calibration total	1.05

**Table 4:** Typical uncertainties in °C for heat pipe blackbody calibration of cableless lightpipe radiation thermometers

Factor	Uncertainty
CLRT noise	0.01
CLRT short-term drift	0.03
Stray radiation	0.00
Blackbody emissivity	0.03
Na-HPBB temperature	0.31

CLRT calibration total	0.31

**Table 5:** Summary of LP measurements in the integrating sphere

Before or after cleaning	LP make	Location		Difference [%]
		Aperture	Inside	
Before	LPRT1-LP2	1.04356 V	1.04803 V	0.43
Before	LPRT2-LP2	1.08129 V	1.09079 V	0.88
After	LPRT1-LP2	1.06788 V	1.06901 V	0.11
After	LPRT2-LP2	1.06654 V	1.06947 V	0.27

**Table 6:** Measurement uncertainties for *in situ* LPRT calibration

Component	*U*/°C
TFTC calibrations	0.4
Thermocouple emf measurements	1.0
LPRT calibrations	0.2
LPRT measurements	0.1
Wafer temperature fluctuations	0.4
Wafer Temperature drift	0.1
Junction/target temperature difference	2.0

Total	2.3

**Table 8:** Temperature uncertainties [°C] for comparison of LPRT and TC measurements

LPRT measurements		TC measurements	
Calibration	0.2	TFTC (10 °C)	0.3
Effective emissivity	3.0	Pd/Pt TC	0.1
Junction/target temperature difference	2.0	TC emf	0.1
Temperature fluctuations	0.4		
Temperature drift	0.1		
LPRT display	0.1		

Subtotal	3.5	Subtotal	0.3

## Footnotes

1 Certain commercial equipment or materials are identified in this paper to foster understanding. Such identification does not imply recommendation or endorsement by the NIST, nor does it imply that the equipment or materials are necessarily the best available for the purpose.
